# Supplementary material for: From sequence to enzyme mechanism using multi-label machine learning
Source: BMC Bioinformatics. 2014 May 19;15:150. doi: 10.1186/1471-2105-15-150 (PMC4229970; doi:10.1186/1471-2105-15-150)
Supplement: Additional file 2 — Java code of ml2db. Additional file ml2db_code.tar.gz contains the Java source code to run the multi-label machine learning experiments and save the results to database. The code’s Javadoc is included. [file 1471-2105-15-150-S2.zip › additional file 2/ml2db/utils/doc/uk/ac/standrews/utils/test/UtilsTest.html]

UtilsTest


JavaScript is disabled on your browser.


- Overview
- Package
- Class
- Use
- Tree
- Deprecated
- Index
- Help

- Prev Class
- Next Class

- Frames
- No Frames

- All Classes

- Summary:
- Nested |
- Field |
- Constr |
- Method

- Detail:
- Field |
- Constr |
- Method


uk.ac.standrews.utils.test

## Class UtilsTest

- java.lang.Object
- - junit.framework.Assert
  - - junit.framework.TestCase
    - - uk.ac.standrews.utils.test.UtilsTest

- All Implemented Interfaces:
  :   junit.framework.Test

  ---

    

  ```
  public class UtilsTest
  extends junit.framework.TestCase
  ```

  Class

  Version:
  :   9 Jul 2008

  Author:
  :   Luna De Ferrari luna.deferrari-at-ed.ac.uk

- - ### Constructor Summary

    Constructors

    Constructor and Description

    `UtilsTest()`
  - ### Method Summary

    Methods

    | Modifier and Type | Method and Description |
    | --- | --- |
    | `void` | `testClass()` public void testBigDecimal(){ this.testBigDecimalGeneric(0.0, 1.0, 0.1); this.testBigDecimalGeneric(0.0, 1.0, 0.2); this.testBigDecimalGeneric(0.0, 1.0, 0.3); } public void testDoubleIncrements1(){ this.testDoubleIncrementsBigDecimal(0.0, 10.0, 0.1); this.testDoubleIncrementsBigDecimal(0.0, 1.0, 0.1); this.testDoubleIncrementsBigDecimal(0.0, 1.0, 0.2); this.testDoubleIncrementsBigDecimal(0.0, 1.0, 0.3); // is not well rounded this.testDoubleIncrementsBigDecimal(0.0, 1.7, 0.3); // increment is null Vector increments = this.testDoubleIncrementsBigDecimal(0.0, 10.0, 0.0); //assertNull(increments); } public Vector testDoubleIncrementsBigDecimal(Double min, Double max, Double percOfIncrement){ TreeSet basicIncrements = Utils.getDoubleIncrementsBigDecimal(min, max, percOfIncrement); if(basicIncrements != null){ Vector increments = new Vector(basicIncrements); Double range = max - min; Double increment = range \* percOfIncrement; if(increment > 0 \|\| increment < 0){ System.out.println("min: " + min + ", max: " + max + ", increment: " + increment + " " + increments.toString()); Double noOfincrements = (range / increment) + 1; int numberOfIncrements = noOfincrements.intValue(); //assertEquals(numberOfIncrements, increments.size()); for(int i=0; i < numberOfIncrements; i++ ){ Double value = min + (i \* increment); //assertEquals(value, (Double) increments.get(i)); } }else{ //assertEquals(null, increments); } return increments; }else{ return null; } } public void testBigDecimalGeneric(Double minDouble, Double maxDouble, Double percentOfIncrementDouble){ BigDecimal min = BigDecimal.valueOf(minDouble); BigDecimal max = BigDecimal.valueOf(maxDouble); BigDecimal percentOfIncrement = BigDecimal.valueOf(percentOfIncrementDouble); Vector intervals = new Vector(); BigDecimal range = max.subtract(min); System.out.println("UtilsTest BigDecimal: " + min + " " + max + " " + percentOfIncrement + " " + range); System.out.println("zero " + BigDecimal.ZERO); assertEquals(1, range.compareTo(BigDecimal.ZERO)); BigDecimal increment = range.multiply(percentOfIncrement); System.out.println("range.divide(increment, RoundingMode.DOWN) " + range.divide(increment, RoundingMode.DOWN)); BigDecimal value = min; value = value.add(increment); System.out.println("value.add(increment) " + value); System.out.println("value.add(increment).ROUND\_DOWN " + value.add(increment).ROUND\_DOWN); System.out.println("max.ROUND\_DOWN " + max.ROUND\_DOWN); System.out.println("---------------------------------"); BigDecimal one0 = BigDecimal.valueOf(1.0); BigDecimal one1 = BigDecimal.valueOf(1.0); BigDecimal range0 = one0.subtract(one1); assertTrue(range0.compareTo(BigDecimal.ZERO) == 0); } |
    | `void` | `testDoubleIncrements2()` |
    | `void` | `testIncrementsUtil()` |
    | `void` | `testIntegerIncrements(java.lang.Integer min, java.lang.Integer max, java.lang.Double incrementAsPercentageOfMaxValue)` |
    | `void` | `testObjectIncrements()` |
    | `void` | `testSeries()` |

    - ### Methods inherited from class junit.framework.TestCase

      `countTestCases, getName, run, run, runBare, setName, toString`
    - ### Methods inherited from class junit.framework.Assert

      `assertEquals, assertEquals, assertEquals, assertEquals, assertEquals, assertEquals, assertEquals, assertEquals, assertEquals, assertEquals, assertEquals, assertEquals, assertEquals, assertEquals, assertEquals, assertEquals, assertEquals, assertEquals, assertEquals, assertEquals, assertFalse, assertFalse, assertNotNull, assertNotNull, assertNotSame, assertNotSame, assertNull, assertNull, assertSame, assertSame, assertTrue, assertTrue, fail, fail, failNotEquals, failNotSame, failSame, format`
    - ### Methods inherited from class java.lang.Object

      `equals, getClass, hashCode, notify, notifyAll, wait, wait, wait`

- - ### Constructor Detail


    - #### UtilsTest

      ```
      public UtilsTest()
      ```
  - ### Method Detail


    - #### testClass

      ```
      public void testClass()
      ```

      public void testBigDecimal(){ this.testBigDecimalGeneric(0.0, 1.0, 0.1);
      this.testBigDecimalGeneric(0.0, 1.0, 0.2);
      this.testBigDecimalGeneric(0.0, 1.0, 0.3); }
      public void testDoubleIncrements1(){
      this.testDoubleIncrementsBigDecimal(0.0, 10.0, 0.1);
      this.testDoubleIncrementsBigDecimal(0.0, 1.0, 0.1);
      this.testDoubleIncrementsBigDecimal(0.0, 1.0, 0.2);
      this.testDoubleIncrementsBigDecimal(0.0, 1.0, 0.3);
      // is not well rounded this.testDoubleIncrementsBigDecimal(0.0, 1.7,
      0.3);
      // increment is null Vector increments =
      this.testDoubleIncrementsBigDecimal(0.0, 10.0, 0.0);
      //assertNull(increments); }
      public Vector testDoubleIncrementsBigDecimal(Double min,
      Double max, Double percOfIncrement){ TreeSet basicIncrements
      = Utils.getDoubleIncrementsBigDecimal(min, max, percOfIncrement);
      if(basicIncrements != null){ Vector increments = new
      Vector(basicIncrements); Double range = max - min; Double
      increment = range \* percOfIncrement;
      if(increment > 0 || increment < 0){ System.out.println("min: " + min +
      ", max: " + max + ", increment: " + increment + " " +
      increments.toString());
      Double noOfincrements = (range / increment) + 1; int numberOfIncrements =
      noOfincrements.intValue();
      //assertEquals(numberOfIncrements, increments.size()); for(int i=0; i <
      numberOfIncrements; i++ ){ Double value = min + (i \* increment);
      //assertEquals(value, (Double) increments.get(i)); }
      }else{ //assertEquals(null, increments); } return increments; }else{
      return null; } }
      public void testBigDecimalGeneric(Double minDouble, Double maxDouble,
      Double percentOfIncrementDouble){ BigDecimal min =
      BigDecimal.valueOf(minDouble); BigDecimal max =
      BigDecimal.valueOf(maxDouble); BigDecimal percentOfIncrement =
      BigDecimal.valueOf(percentOfIncrementDouble);
      Vector intervals = new Vector(); BigDecimal range
      = max.subtract(min);
      System.out.println("UtilsTest BigDecimal: " + min + " " + max + " " +
      percentOfIncrement + " " + range); System.out.println("zero " +
      BigDecimal.ZERO); assertEquals(1, range.compareTo(BigDecimal.ZERO));
      BigDecimal increment = range.multiply(percentOfIncrement);
      System.out.println("range.divide(increment, RoundingMode.DOWN) " +
      range.divide(increment, RoundingMode.DOWN)); BigDecimal value = min;
      value = value.add(increment); System.out.println("value.add(increment) "
      + value); System.out.println("value.add(increment).ROUND\_DOWN " +
      value.add(increment).ROUND\_DOWN); System.out.println("max.ROUND\_DOWN " +
      max.ROUND\_DOWN); System.out.println("---------------------------------");
      BigDecimal one0 = BigDecimal.valueOf(1.0); BigDecimal one1 =
      BigDecimal.valueOf(1.0); BigDecimal range0 = one0.subtract(one1);
      assertTrue(range0.compareTo(BigDecimal.ZERO) == 0);
      }


    - #### testDoubleIncrements2

      ```
      public void testDoubleIncrements2()
      ```


    - #### testIncrementsUtil

      ```
      public void testIncrementsUtil()
      ```


    - #### testIntegerIncrements

      ```
      public void testIntegerIncrements(java.lang.Integer min,
                               java.lang.Integer max,
                               java.lang.Double incrementAsPercentageOfMaxValue)
      ```


    - #### testObjectIncrements

      ```
      public void testObjectIncrements()
      ```


    - #### testSeries

      ```
      public void testSeries()
      ```


- Overview
- Package
- Class
- Use
- Tree
- Deprecated
- Index
- Help

- Prev Class
- Next Class

- Frames
- No Frames

- All Classes

- Summary:
- Nested |
- Field |
- Constr |
- Method

- Detail:
- Field |
- Constr |
- Method
